# Natural variation of *Arabidopsis thaliana* responses to *Cauliflower mosaic virus* infection upon water deficit

**DOI:** 10.1371/journal.ppat.1008557

**Published:** 2020-05-15

**Authors:** Sandy E. Bergès, François Vasseur, Alexis Bediée, Gaëlle Rolland, Diane Masclef, Myriam Dauzat, Manuella van Munster, Denis Vile

**Affiliations:** 1 LEPSE, Univ Montpellier, INRAE, Montpellier SupAgro, Montpellier, France; 2 BGPI, Univ Montpellier, CIRAD, INRAE, Montpellier SupAgro, Montpellier, France; 3 CEFE, CNRS, EPHE, IRD, Univ Montpellier, Univ Paul Valéry Montpellier, Montpellier, France; The Ohio State University, UNITED STATES

## Abstract

Plant virus pathogenicity is expected to vary with changes in the abiotic environment that affect plant physiology. Conversely, viruses can alter the host plant response to additional stimuli from antagonism to mutualism depending on the virus, the host plant and the environment. Ecological theory, specifically the CSR framework of plant strategies developed by Grime and collaborators, states that plants cannot simultaneously optimize resistance to both water deficit and pathogens. Here, we investigated the vegetative and reproductive performance of 44 natural accessions of *A*. *thaliana* originating from the Iberian Peninsula upon simultaneous exposure to soil water deficit and viral infection by the *Cauliflower mosaic virus* (CaMV). Following the predictions of Grime’s CSR theory, we tested the hypothesis that the ruderal character of a plant genotype is positively related to its tolerance to virus infection regardless of soil water availability. Our results showed that CaMV infection decreased plant vegetative performance and annihilated reproductive success of all accessions. In general, water deficit decreased plant performance, but, despite differences in behavior, ranking of accessions tolerance to CaMV was conserved under water deficit. Ruderality, quantified from leaf traits following a previously published procedure, varied significantly among accessions, and was positively correlated with tolerance to viral infection under both well-watered and water deficit conditions, although the latter to a lesser extent. Also, in accordance with the ruderal character of the accession and previous findings, our results suggest that accession tolerance to CaMV infection is positively correlated with early flowering. Finally, plant survival to CaMV infection increased under water deficit. The complex interactions between plant, virus and abiotic environment are discussed in terms of the variation in plant ecological strategies at the intraspecific level.

## Author summary

Virus pathogenicity may be influenced by changes in the abiotic environment. A common change is decrease in soil water availability, which is detrimental to plant productivity and the occurrence of which is expected to increase due to climate change, has recently been shown to interfere with plant–virus interactions. We investigated the performance of 44 natural accessions of the plant species *Arabidopsis thaliana* infected by *Cauliflower mosaic virus* under well-watered and water deficit conditions. We showed that viral infection decreased plant vegetative performance and annihilated reproductive success of all accessions, and that these pathogenic effects were increased by water deficit. Intrinsic characteristics of the accessions were related to their tolerance to the virus so that accessions with low leaf tissue density and rapid growth rate were more tolerant to viral infection regardless of watering condition. Finally, plant survival upon viral infection increased under water deficit. We discuss the role of intrinsic plant characteristics, seen as ecological strategies, in plant tolerance to viral infections under contrasting environmental conditions, and the consequences for the study of viral epidemiology.

## Introduction

A greater understanding of the outcome of plant-virus interactions under contrasting environmental conditions is urgently required given current global climate change conditions [1]. Indeed, to ensure their survival and successful reproduction, plants must be able to respond appropriately and effectively to biotic and abiotic changes in their environment [2]. However, interactions between plant responses to drought and pathogen virulence or detrimental effects on host performance remain poorly known. In the present study, we analyzed the outcome of the interaction between water deficit and viral infection, and tested to what extent plant tolerance to these factors, as quantified by biomass reduction, is related to intraspecific variations in plant ecological strategies.

Infection by pathogenic viruses is a major biotic constraint that strongly impacts plant performance at both the vegetative and reproductive levels. However, the success of viral infection, and its quantitative variation of pathogenicity depends on the physiological machinery of the host plant, and thus any other environmental change that affects plant physiology may also affect the outcome of viral infection [3]. Conversely, it has been shown that viruses can alter the host plant’s response to additional stimuli, and that the outcome of this response can vary from antagonism to mutualism depending on the virus, the host plant and the environment [4–7]. In particular, limiting soil water availability is an increasingly recurrent condition affecting plant physiology and represents a major constraint to plant growth and productivity [8,9]. Depending on the specific drought scenario, and the time of its occurrence during their life cycle, plants combine different response strategies [9,10] driven by intricate regulatory networks [11], and these strategies may also interact with virus infection [12–14]. Plant responses to water deficit might also have additional side effects on plant resistance to pathogens.

In recent decades, comparative approaches in trait-based ecology have identified a set of key plant functional traits that reflect differences in ecological strategies between and within species [15–17]. Among the different frameworks proposed to evaluate these differences, Grime’s CSR triangle theory [18] has been outstanding, even at the intraspecific level, where natural selection basically operates [19,20]. This theory states that there are two main environmental factors that drive plant diversification: (i) disturbance, i.e. any environmental factor that causes partial or total destruction of plant biomass, which includes pathogens, grazing, trampling and mowing; and (ii) stress, i.e. environmental factors that reduce plant growth because of resource shortage [18]. Grime’s theory also posits that plants cannot simultaneously optimize the response to both disturbance and stress [18]. As a consequence, variation in disturbance and stress intensity are expected to coincide with quantitative variation in three main plant ecological strategies: (1) competitors (C) in highly productive habitats with low stress intensity and disturbance, which invest resources in the rapid growth of large organs to outcompete neighbors; (2) stress-tolerance (S) in stressed, resource-poor habitats with low disturbance, which invest resources to protect tissue from stress damages; and (3) ruderals (R) in resource-rich environments associated with repeated disturbance, which invest resources in rapid reproduction and propagule dispersal [18,21,22]. Correlatively to Grime’s theory expectations, it suggests that plants cannot optimize tolerance to both pathogen infection and drought. However, these expectations remain theoretical thus far and need to be tested experimentally.

In Grime’s framework, resistance to pathogens must be a characteristic of ruderality (R), which is associated with short-lived plants that reproduce quickly and possess thin leaves with high nutrient concentration and high net photosynthetic rate [18,23]. By contrast, stress tolerance (S), notably drought tolerance, must be associated with long-lived plants with small, tough leaves, low nutrient concentration and low net photosynthetic rate [18,22]. Indeed, reduced growth rate and subsequent total leaf area advantageously reduce potential loss of water through transpiration, and thus are part of the water saving strategy [2,24]. Consistently, three leaf traits are commonly used as indicators of CSR strategies: leaf area; the ratio of leaf dry mass to leaf area (LMA); and leaf dry matter content (LDMC), i.e. the ratio of leaf dry mass to leaf water-saturated fresh mass. These traits are easily measurable and are related to key leaf and whole-plant physiology markers, such as net photosynthetic rate and water loss through transpiration [17,20,25–27]. In addition, plant development and phenology are key components of the strategies to cope with pathogen infection and water stress. In annual plants, reduced growth rate and delayed reproduction are often reported in response to moderate water deficit. However, in some instances such as a severe or sudden water deficit, increased growth rate and hastened reproduction can be observed and interpreted as an escape strategy related to plant ruderality [11].

In contrast with Grime’s expectations, the effects of water deficit on plant development and leaf physiology may reduce virus systemic spread, and therefore decrease virulence [13,28,29]. Virus-induced drought tolerance has been demonstrated in several species infected with different viruses [4,30–33]. Recent findings in *Arabidopsis thalian*a demonstrated that spread of *Cauliflower mosaic virus* (CaMV; Caulimoviridae, a non-circulative virus transmitted by aphids that infects essentially plants of the family Brassicaceae) infection throughout the host plant was slower under water deficit, though the combined effects of CaMV infection and water deficit remained more detrimental to growth compared with either viral infection or water deficit alone [13]. In the present study, we investigated the effects of water deficit and infection by CaMV on the vegetative and reproductive performance of the wild plant *A*. *thaliana* (Brassicaceae) in order to test Grime’s theoretical expectations against potential alternative outcomes of drought and pathogen interactions.

*A*. *thaliana* is an annual species that occurs in a large range of climates [34] and that is increasingly used as a model for analyses of evolutionary ecology of plant-parasite interactions [35–37]. Like most annual plants, this rosette-shaped species reproduces quickly and invests resources preferentially in the production and dispersal of propagules [38]. It is commonly found in disturbed habitats, and is thus considered as a mostly ruderal species [18]. However, *A*. *thaliana* exhibits significant intraspecific trait variation among accessions [39–42] that translates into significant variation in ecological strategies [43], especially along the ruderality (R) axis of Grime’s CSR framework [19,20,27]. Here, we examined the effects of CaMV infection in a set of 44 natural *A*. *thaliana* accessions originating from the Iberian Peninsula. We focused on the Iberian Peninsula because (i) this region is characterized by a wide range of climatic variations, notably in terms of annual precipitation, with severe summer drought in the South and mesic conditions in the North; and (ii) CaMV has been found to infect natural Spanish populations of *A*. *thaliana* [36]. In addition, significant variation in tolerance to infection by CaMV has been found previously among *A*. *thaliana*’s ecotypes [13]. For instance, virulence of CaMV was reduced in early flowering ecotypes [44]. Consistently, genotypes of *A*. *thaliana* with inherently longer life span were also more tolerant to infection by *Cucumber mosaic virus* (CMV) [35,45,46]. To test Grime’s theoretical expectations, we quantified morpho-physiological traits and ecological strategies under strictly controlled environmental conditions in the high throughput phenotyping platform PHENOPSIS [47]. We then tested to what extent plant tolerance, as quantified by the relative change of aboveground dry mass, to viral infection and water deficit are related to these traits and ruderality. Specifically, we tested the hypothesis that ruderality is positively related to plant tolerance to virus infection whatever the conditions of water availability.

## Results

### *Arabidopsis thaliana* growth response to CaMV infection and water deficit

**Fig 1:**
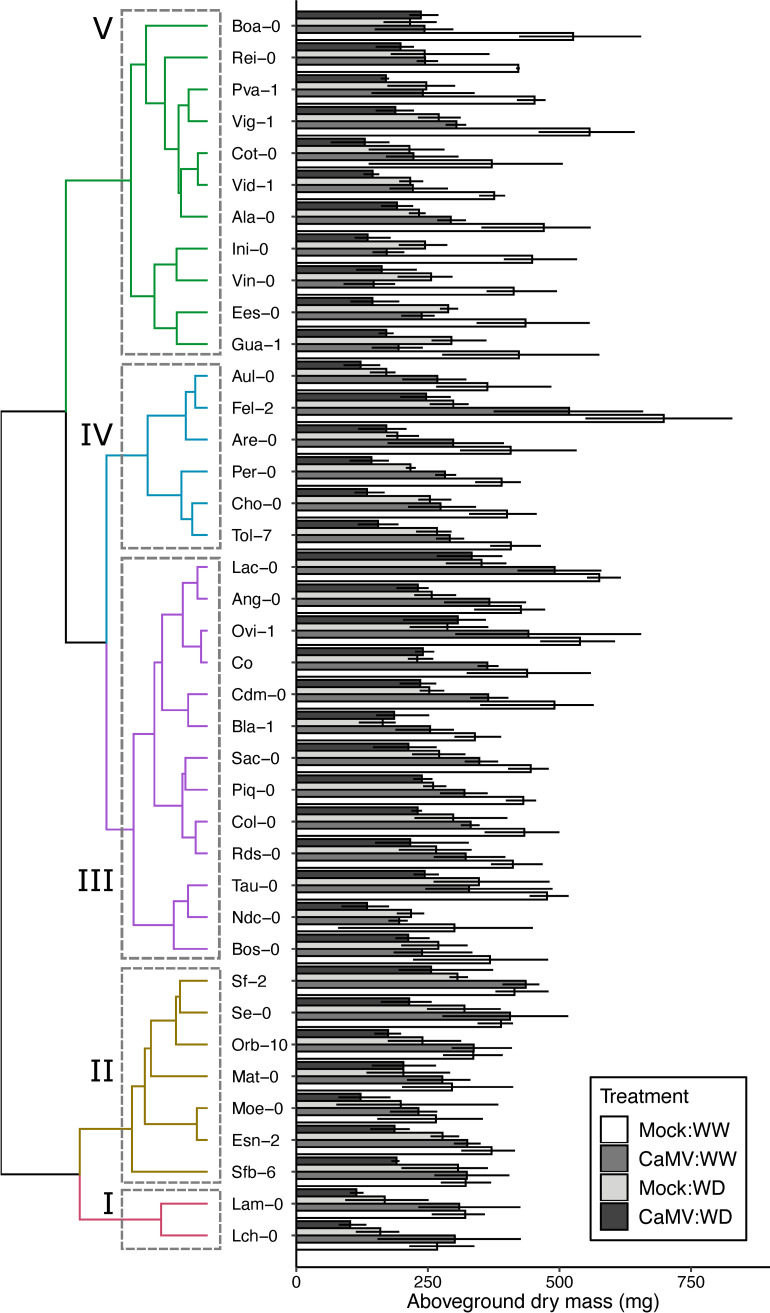
Effects of CaMV infection and watering on growth of 39 accessions of *A*. *thaliana*. (Left) Clustering based on joint Euclidean distance of the changes in aboveground dry mass (%) in response to infection with CaMV, soil water deficit (WD) and combination of both treatments, relatively to mock-inoculation under well-watered (WW) conditions. Color of the branches in the dendrogram highlights five clusters of accessions with similar responses. (Right) Bars are mean ± bootstrapped 95% CI of aboveground dry mass at 30 dpi (n = 3) of plants grown in mock-inoculated:WW (white bars), CaMV-infected:WW (dark grey bars), mock-inoculated:WD (light grey bars) and CaMV-infected:WD (black bars) conditions. Data are from Experiment 1.

Across the 44 Iberian natural accessions (S1 Table; S1 Fig), no significant relationship between climate or altitude and the traits measured in this study was found. CaMV successfully infected plants of all accessions. A highly significant variation of aboveground dry mass, measured on individual plants 30 days after CaMV- or mock-inoculation, under well-watered and water deficit conditions, was found among accessions (Fig 1; S2 Table; n = 39; *P* < 0.001; Experiment 1). Under well-watered conditions, mean aboveground dry mass (± sd) ranged from 267 ± 36 mg (Lch-0) to 697 ± 81 mg (Fel-2) (Fig 1). This variation reflected the strong differences in growth rate among accessions (S2 Fig). Moreover, a highly significant variation in accession responses to CaMV infection and water deficit were found (both *P* < 0.001; S2 Table), but no significant interactive effect between accession and watering nor between inoculation and watering was detected (*P* = 0.47 and *P* = 0.36, respectively; S2 Table; S3 Fig).

**Fig 2:**
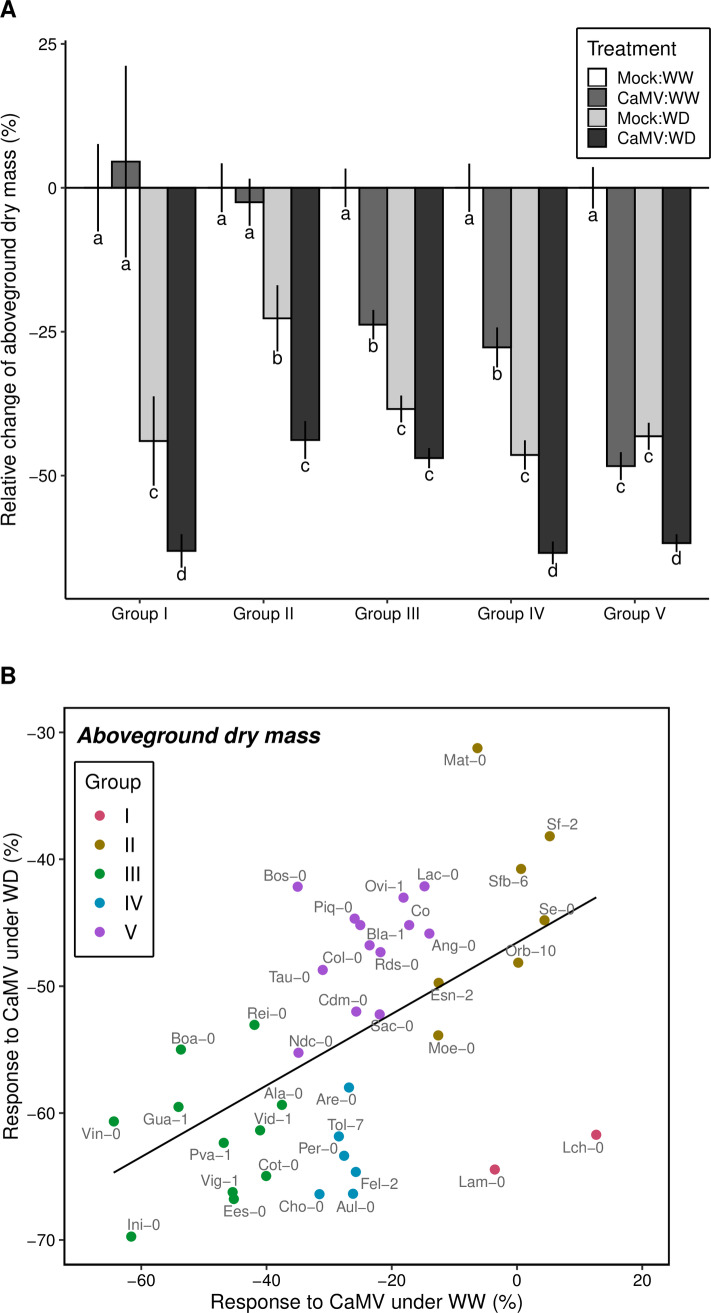
Relative change of aboveground dry mass in five groups of accessions responses to CaMV infection and watering treatment. (A) Means (± se) of relative change of aboveground dry mass for each response group in Fig 1 under mock-inoculated:WW (white bars), CaMV-infected:WW (dark grey bars), mock-inoculated:WD (light grey bars) and CaMV-infected:WD (black bars) conditions. Different letters indicate significant differences at the 5% probability level using Tukey adjustment method. (B) Relationship between accessions response to CaMV infection under WW and WD conditions. Each point represents an accession from the five response groups (colors are similar to Fig 1). Line represents significant linear regression at *P* < 0.05. WW: well-watered; WD: water deficit. Data are from Experiment 1.

Tolerance of *A*. *thaliana* to water deficit and CaMV infection was quantified as relative change of mean aboveground dry mass of the rosette. Clustering based on conjoint responses of aboveground dry mass to all treatments revealed five major groups of accessions (Figs 1 and 2). Accessions of groups I and II were particularly tolerant, i.e. exhibited a lower relative reduction of aboveground dry mass, to CaMV infection, whereas those of groups III and IV had intermediate tolerance and those of group V were the most sensitive to CaMV infection. CaMV infection did not significantly affect dry mass production of accessions from groups I and II (all *P* > 0.05), although these plants showed clear viral symptoms. Accessions of all groups exhibited a similar decrease in dry mass in response to water deficit but those of group III had intermediate tolerance and accessions of group II were the most tolerant to water deficit (all *P* > 0.08). Accessions from group I and III were strongly sensitive to water deficit, but accessions from group I were also characterized by comparatively higher tolerance to CaMV (Figs 1 and 2).

Variation of accession tolerance/susceptibility to both CaMV and water deficit translated into a significantly positive correlation between changes in aboveground dry mass in response to each stress (*r* = 0.39; *P* = 0.013; S4A Fig). Overall, the combination of water deficit and viral infection tended to be more deleterious than each of the two stresses taken separately. Aboveground dry mass decreased significantly in all accessions in response to the combination of water deficit and viral infection (Fig 1; all *P* < 0.05 but Mat-0, *P* = 0.23), and the most tolerant accessions clustered in groups II and III (Fig 2). The response varied from 30% to 70% reduction of aboveground dry mass in Mat-0 and Ini-0 (Fig 1, S4B Fig), respectively. The contrasted levels of tolerance led to significant positive correlations between CaMV infection or water deficit alone and combined stress conditions across accessions (*r* = 0.54, *P* < 0.001 and, *r* = 0.41, *P* = 0.003, respectively; Fig 2B, S4 Fig). In other words, in terms of dry mass accumulation, accessions that were tolerant (or susceptible) to CaMV infection were also tolerant (or susceptible) to the double stress (Fig 2). This observation is exemplified in accessions of group II (Fig 1) which were particularly tolerant to CaMV infection, and remained CaMV-tolerant under water deficit, while accessions from group V were highly susceptible to CaMV infection, independently of the watering conditions (S4 Fig). By contrast, accessions from group III and IV had similar tolerance to CaMV under well-watered conditions, but contrasted responses to the virus under water deficit that was apparently not explained by the similarity of response to water deficit (S4 Fig). Accessions from group I were highly tolerant to CaMV infection under well-watered conditions, but were highly susceptible when water deficit was applied in addition to CaMV infection (65% reduction of aboveground dry mass; both *P* < 0.05). Several other accessions had increased susceptibility to CaMV infection upon water deficit that was not explained by the response to water deficit alone, i.e. aboveground dry mass under water deficit was more affected in CaMV-infected than in mock-inoculated plants. As shown by the position of the accessions under the line of equal response, we found no significant positive effect of CaMV infection on plant tolerance to water deficit in terms of aboveground dry mass (S4B Fig).

### Co-variations in leaf functional traits upon combined virus infection and water deficit

Leaf mass per area (LMA) and leaf dry matter content (LDMC) were significantly correlated and exhibited significant variation among accessions both in means and in relative responses to CaMV, water deficit, and their combination (S5 and S6 Figs; S2 Table). Both leaf traits generally increased in response to the treatments (S5 and S6 Figs), i.e. leaves were denser and/or thicker, and/or had lower water content in response to CaMV infection, water deficit, or both.

A significant and positive correlation was detected between the responses of LMA and LDMC to CaMV infection in each watering treatment (*r* = 0.87, *P* < 0.001 for well-watered and *r* = 0.83, *P* < 0.001 for water deficit). However, the slopes of the regressions were significantly different (*P* < 0.001) due to larger changes in LDMC compared with changes in LMA. Indeed, we found that the relative responses of LMA to CaMV under well-watered and water deficit conditions were significantly correlated (*r* = 0.68, *P* < 0.001). In other words, water deficit did not perturbate by too much the ranking of changes in LMA due to CaMV infection among accessions.

### Accession tolerance to viral infection increases with ruderality

To determine the relationship between tolerance to CaMV-infection and water deficit and functional strategies of the accessions, we tested the relationships between changes in aboveground dry mass in response to the treatments and the ruderality index (R) obtained from C:S:R scores calculated from leaf traits of healthy plants under well-watered conditions. We first compared the correlations of growth rate and leaf traits of the accessions at 8 and 12-h day lengths to test the generality of the relationship (S7 Fig). Correlations between LMA and LDMC were highly significant and similar at both day lengths (*r* = 0.87 and *r* = 0.88, both *P* < 0.001 respectively; S7A and S7B Fig), with no, or only marginally significant, interaction effect of CaMV infection and watering treatment (S3 Table). The maximum rate of leaf expansion of the rosettes was different among accessions and between experiments (S7C Fig; n = 15). However, a highly significant positive correlation was found between the two experiments (*r* = 0.71; *P* = 0.003; S7C Fig). The ranking between experiments was significantly conserved for LMA and LMDC when four accessions susceptible to photoperiod (Orb-10, Vig-1, Lam-0, Moe-0) were excluded from the analyses (*r* = 0.47, *P* = 0.001; *r* = 0.48, *P* = 0.001, respectively; n = 43).

**Fig 3:**
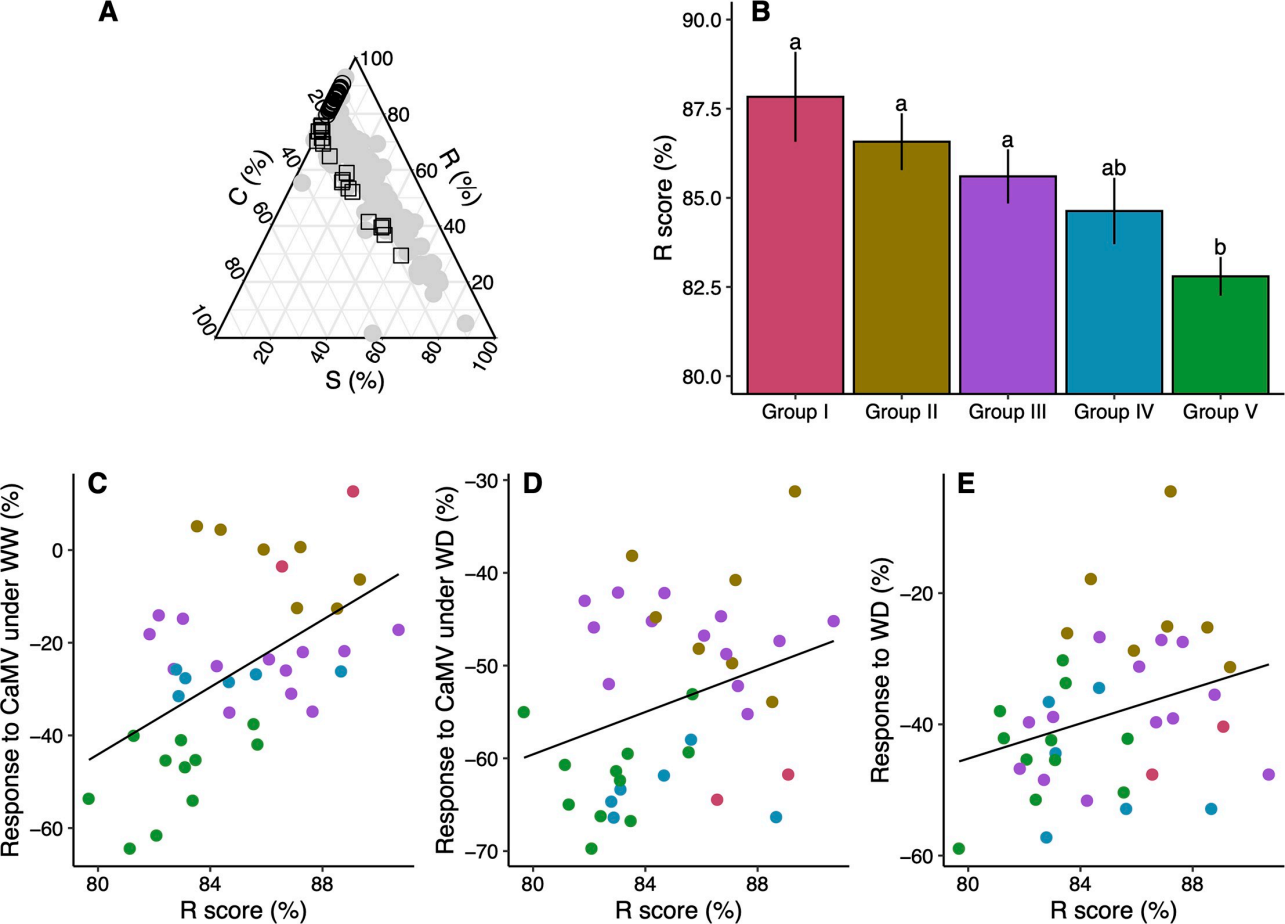
Grime’s CSR framework and response of *A*. *thaliana* accessions to CaMV infection water deficit. (A) CSR representation of 44 accessions from two independent experiments (Experiment 1, n = 39, 8-h day length, circles; Experiment 2, n = 20, 12-h day length, squares). Grey circles represent the data extracted from [20]. (B) Mean (± se) ruderality (R, %) score of accessions from the respective response groups in Fig 1. Different letters indicate significant differences at the 5% probability level using Tukey adjustment method. (C-E) Relationships between accessions’ R-score and relative change of aboveground dry mass of CaMV-infected plants under well-watered (WW) and water deficit (WD) conditions, and of mock-inoculated plants grown under WD. Each point represents an accession. Colors are similar to groups in Fig 1. Lines represent significant linear regressions: (C) *r* = 0.53, *P* < 0.001; (D) *r* = 0.31, *P* = 0.052; (E) *r* = 0.31, *P* = 0.053.

The pattern of variation of Grime’s CSR functional strategies across accessions was similar to that found in a larger set of *A*. *thaliana*’s natural accessions (Fig 3A). As expected for an annual ruderal species, all accessions had high R scores (mean ± SE = 85.1 ± 0.43%) ranging from 80.2 to 90.8% (open circles in Fig 3A). Noticeably, average R score rank decreased in order from group I to group IV (Fig 3B). Therefore, under well-watered conditions, position of an accession along the R-axis was positively correlated with plant tolerance to CaMV infection (*r* = 0.53, *P* < 0.001; Fig 3B; S5 Fig). Under water deficit, a positive but weaker relationship was also found (*r* = 0.31, *P* = 0.052; Fig 3B; S5 Fig). Finally, the R-score was marginally positively correlated with plant tolerance to water deficit (*r* = 0.31, *P* = 0.053; Fig 3C; S5 Fig). Thus, the ruderality of an accession was positively correlated to its capacity to tolerate CaMV infection and, to a lesser extent, water deficit. Accordingly, negative correlations were found between LDMC determined under control conditions and the change in aboveground dry mass due to CaMV infection (*r* = –0.63, *P* < 0.001; S9A Fig). Under water deficit, LDMC was negatively correlated with change in aboveground dry mass at the whole-plant (*r* = –0.41, *P* < 0.008), but not at the leaf, level (*r* = –0.20; *P* = 0.23; S9A Fig). Similar negative correlations were found between LMA and change in aboveground dry mass (S9B Fig). Therefore, accessions with lower leaf tissue density and/or thinner leaf lamina were more tolerant to CaMV infection. We then investigated the relationships between leaf traits, R-scores and the timing of reproduction (determined at 12-h day length; S10 Fig). Bolting time and flowering time were both strongly positively correlated with LDMC (*r* = 0.91, *P* < 0.001; S11A Fig and *r* = 0.89, *P* < 0.001; S11D Fig, respectively). Similar positive correlations were found with LMA (*r* = 0.89, *P* < 0.001; S11B Fig and *r* = 0.84, *P* < 0.001; S11E Fig, respectively). Moreover, accession’s R-score was significantly negatively correlated with days to bolting and days to flowering (*r* = –0.90, *P* < 0.001; S11C Fig and *r* = –0.88, *P* < 0.001; S11F Fig, respectively). Thus, ruderality of an accession, as determined in its vegetative phase, was positively correlated to early reproduction.

### Water deficit reduces, and CaMV infection annihilates, the reproductive success of *A*. *thaliana*

**Fig 4:**
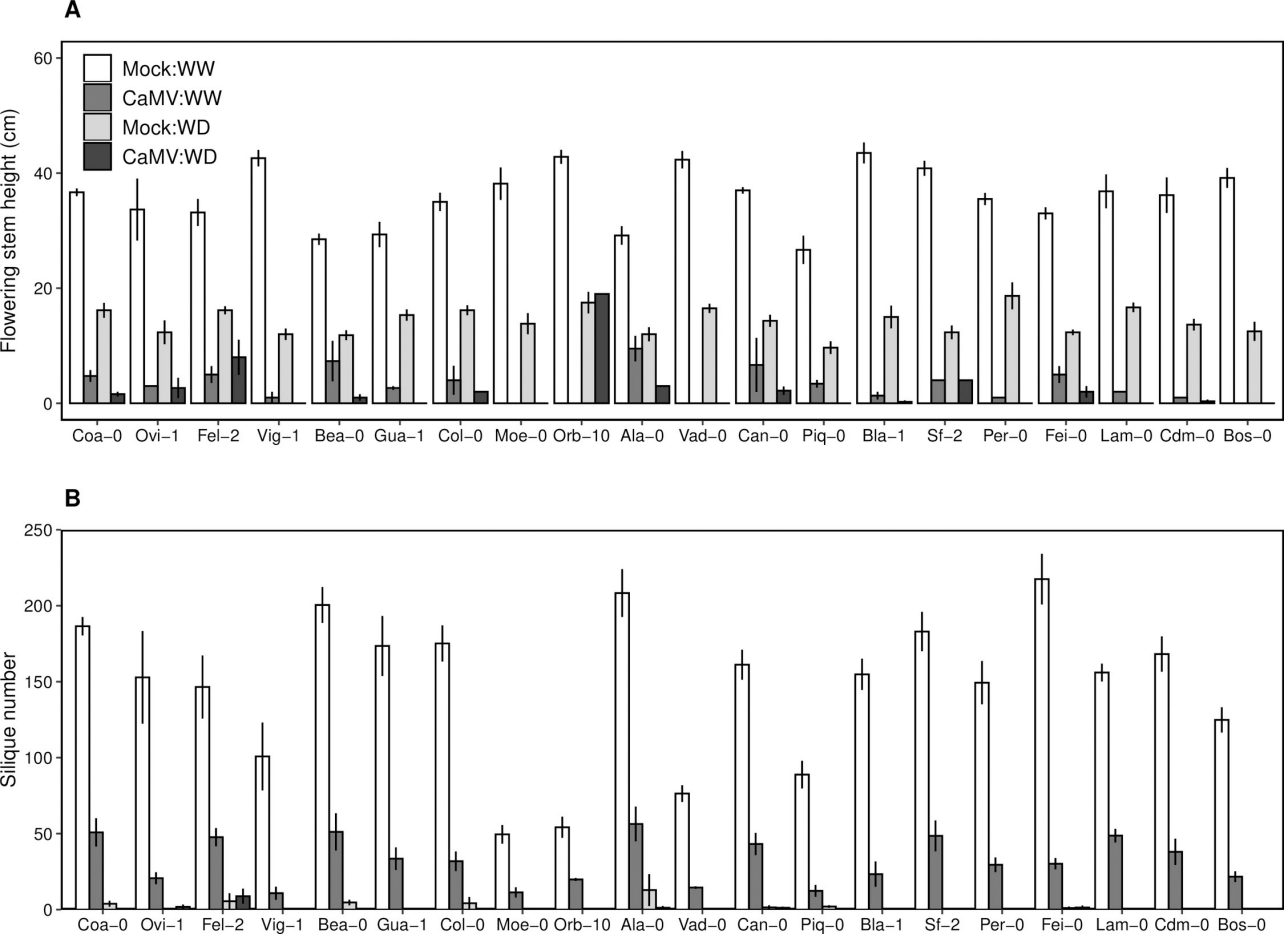
Effects of CaMV infection and watering treatment on reproductive traits of 20 *A*. *thaliana* accessions. (A) Flowering stem height (cm). (B) Silique number. Bars and error bars are means ± bootstrapped 95% confidence intervals of plants grown under mock-inoculated:WW (white bars), mock-inoculated:WD (dark grey bars), CaMV-infected:WW (light grey bars) and CaMV-infected:WD (black bars) conditions. Accessions are ordered according to final projected area of the rosette in condition of viral infection. WW: well-watered; WD: water deficit. Data are from Experiment 2.

Individual reproductive success was estimated by the length of the main flowering stem and the number of mature siliques in 20 accessions (Experiment 2). Both traits were reduced strongly upon water deficit, and they were even more impacted when plants undergo CaMV infection (Fig 4). Under water deficit, stem length was reduced by, on average, 50% in all accessions, corresponding to a strong reduction (50–80%) of silique number (Fig 4). When infected with CaMV, even though some accessions produced very small flowering stems (e.g. Orb-10, Ovi-0, Col-0, Ala-0), no silique, and therefore no seeds, were produced (Fig 4).

### Dynamics of viral symptoms can be modified by soil water deficit

To determine the effects of the watering regime on CaMV symptom development, we estimated the timing of symptom appearance, the rate of systemic spread, and the maximum proportion of infected plants in 20 accessions. The proportion of CaMV-inoculated plants showing characteristic virus symptoms varied from 50% to 100% across accessions whatever the soil watering treatment. In well-watered conditions, the mean time of symptom appearance on the first non-inoculated leaf was estimated at 11.7 days post inoculation (S12A Fig). However, the lag time to symptom appearance (± 95% CI) varied significantly between accessions, from 9.7 ± 0.3 dpi in Cdm-0 to 16.1 ± 1.0 dpi in Bea-0 (*P* < 0.05). Lag time to symptom appearance in response to water deficit decreased significantly (i.e. faster appearance of first symptoms) in seven accessions, whereas it increased significantly in five others (S12A Fig). There was no significant effect of water deficit on this parameter on the eight remaining accessions (S12A Fig). The rate of systemic spread decreased significantly under water deficit compared with well-watered conditions in six accessions, while it increased in six others (S12B Fig; *P* < 0.05). In the eight last remaining accessions no effect of water deficit on the rate of systemic spread was observed (S12B Fig).

### Survival of CaMV infected plants is improved under water deficit

**Fig 5:**
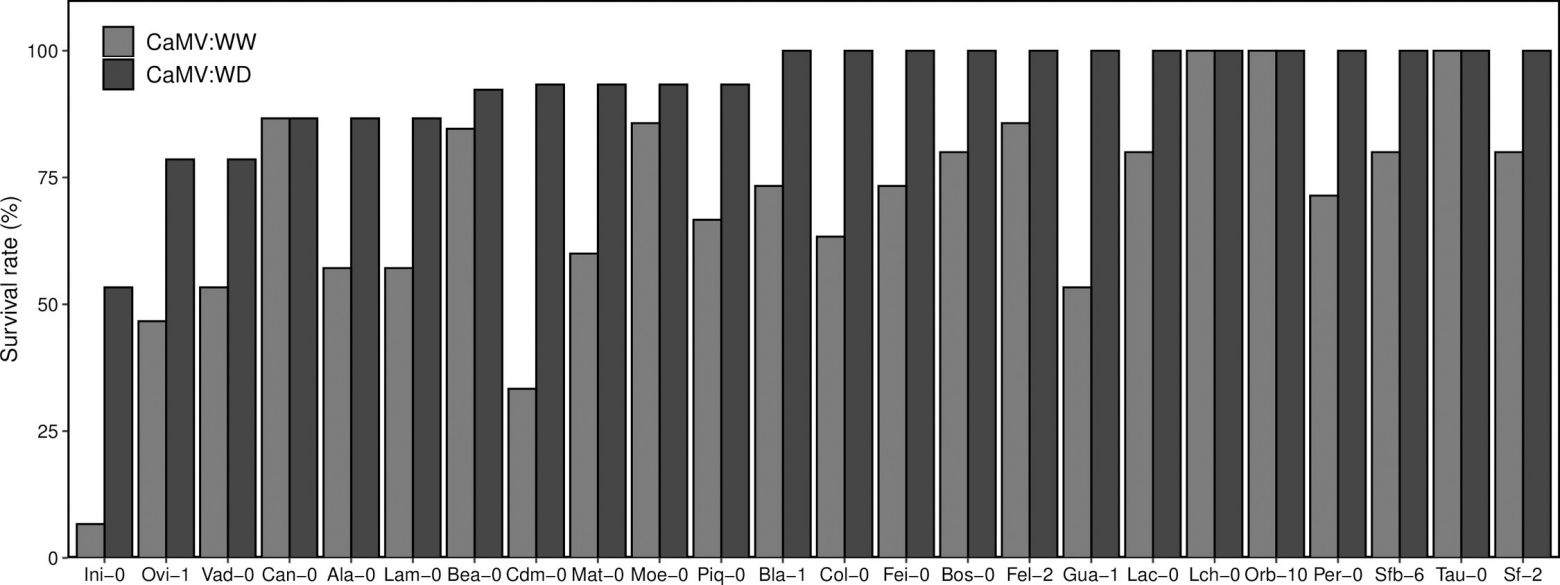
Survival rate of 20 accessions of *A*. *thaliana* infected with CaMV under well-watered and water deficit conditions. Plant survival in response to CaMV infection under well-watered (WW, grey bars) and water deficit (WD, black bars) conditions as determined in Experiment 2 30 days post inoculation. Accessions are ordered according to increasing % of survival of CaMV-infected plants under water deficit.

Survival of CaMV-infected plants (n = 24) was estimated for each accession from the ratio of dead CaMV-infected plants to the total number of infected plants (%) for each watering condition. At a given time-point (30 days after germination), we observed, for almost all accessions, that survival of CaMV-infected plants was higher when plants were cultivated under water deficit than when they were cultivated under well-watered conditions. For instance, survival rate of Ini-0 was 10% under well-watered conditions but increased to 55% under water deficit, and survival increased from 30% to 95% for Cdm-0 (Fig 5). In three accessions (Lch-0, Orb-10 and Tau-0), all plants survived regardless of watering conditions.

## Discussion

We evaluated the performance of 44 wild plant accessions of *A*. *thaliana* originating from the Iberian Peninsula to simultaneous exposure to water deficit and viral infection by CaMV, a virus that naturally infects *A*. *thaliana*'s populations in this geographic area. To this purpose, various phenological, growth and morpho-physiological traits were analyzed to determine plant performance in the vegetative and reproductive phases. We tested the hypothesis that plant tolerance to a viral infection or combined stresses is related to inherent (i.e. determined under control, non-stressing conditions) trait values of the accessions and their covariations, i.e. plant functional strategies. More precisely, following the theoretical predictions of Grime’s triangle of CSR strategies [18], we tested the hypothesis that ruderal character is positively related to plant tolerance to virus infection, whatever the condition of water availability. Following Grime’s definition, ruderals are plant species from resource-rich environments associated with repeated disturbance, that invest resources in rapid reproduction and propagule dispersal [18]. Although CSR strategies have been seminally defined at the interspecific level, recent studies have demonstrated that intraspecific variation is under genetic determinism and is ecologically grounded [19,20,27].

Here, we have shown that CaMV infection modifies morpho-physio-phenological traits of all accessions, affects plant vegetative performance and, annihilates plant reproductive success. In general, water deficit decreased plant performance and despite differences in behavior among accessions, we found a conserved ranking of accession tolerance to CaMV when combined with water deficit. We followed the procedure of [23] to determine the position of each accession in the CSR triangle from three easily measurable traits, namely leaf area, LDMC, and LMA. We first showed that position in the CSR triangle was in accordance with recent findings [20,27]. Second, we found that tolerance to viral infection was correlated positively with position along the ruderality (R) axis under well-watered conditions and, to a lesser extent, upon water deficit. Also, in accordance with the ruderal character of the accession, and previous findings [13,48], we showed that tolerance is likely positively correlated with early flowering. We investigated the relationships between climate at the collection sites (obtained from WorldClim v. 1.4, http://worldclim.org) and accession responses to both stresses, but no significant relationships were found. Further studies will be required to investigate the relationship between response to viral infections and local environmental conditions.

### Natural accessions cluster in a limited number of responses to CaMV, water deficit, and their combination

In accordance with our previous findings in nine Eurasian *A*. *thaliana* accessions infected by CaMV [13], we observed a significant variation in tolerance to CaMV and water deficit among the 44 Iberian accessions. The combination of viral infection and water deficit was even more detrimental to plant growth compared to our previous study (10–40% in [13] and 30–70% here) and in most cases compared with each stress individually. Although some combinations of stresses have been shown to be additive [49], interactive effects between biotic and abiotic stresses are most frequently reported [1,2,29,46,50]. In contrast to our previous findings [13], we found here a weak but significantly positive correlation between tolerance to CaMV infection and tolerance to water deficit (S4 Fig). Differences in experimental conditions and increased number of assayed plants accessions may explain this discrepancy. This result may be in accordance with previous studies that reported similar hormonal, molecular or biochemical pathways induced in plants by viral infection or a water deficit [51–54]. This may also indicate that the ability of a genotype to endure a stress is part of its inherent functional strategy, at least partly independently of the type or level of stress considered (see below). However, the weakness of the relationship suggests strong genotypic variation of plant responses to both stresses and/or that different mechanisms are also involved. In addition, we found a positive correlation between tolerance to CaMV determined upon well-watered and water deficit conditions. Indeed, water deficit did not affect too much the ranking of changes in aboveground dry mass due to CaMV infection among accessions.

Considering the growth responses to CaMV infection, water deficit and their combination together, we identified five clusters of accessions. Some accessions (group III) were mildly susceptible to CaMV infection while highly susceptible to water deficit. In such cases, the effect of combined stresses was similar to that of water deficit alone. Other accessions of group IV, such as Tol-7 or Cho-0, had a similar susceptibility to water deficit or CaMV, but the combination of both stresses raised susceptibility level. A third group (group V) enclosed accessions with high susceptibility to both CaMV or water deficit but the effect of their combination was close to one or both of the individual stresses. Accessions in the group II were particularly tolerant to CaMV infection, not very susceptible to water deficit, but susceptible to stress combination. Finally, two accessions, Lch-0 or Lam-0 (group I) were also tolerant to CaMV, but more susceptible to water deficit, and even more susceptible to the double stress. This may illustrate the limited diversity of plant responses that could be encountered under natural conditions where combinations of stresses are common, and may open avenues to develop modeling approaches when limited knowledge on genotype-specific response is available.

### Survival, but not reproductive effort, of infected plants is improved by water deficit

Pagán et al. (2008) showed that tolerance to *Cucumber mosaic virus* (CMV) in the vegetative phase was also associated with increased seed production and a shortened reproductive period, reducing the time span between the production of reproductive structures and seed production. By contrast, Shukla et al. (2018) recently showed that CMV-tolerant accessions did not show similar resource reallocation patterns in response to more virulent viruses, suggesting that plant tolerance is virus-specific. Here, we found that performance upon CaMV infection in the vegetative phase was not related to performance in the reproductive phase, since none of the plants developed a mature silique, and therefore no seeds were produced.

Several studies have reported increased plant tolerance to abiotic stresses, including water deficit, upon infection by viruses [4,30,32]. Across the quite large number of plant accessions assayed here (n = 44), we did not find any significant positive effect of CaMV infection on plant tolerance to water deficit in terms of vegetative biomass production (see position of accessions relative to line of equal response on S4B Fig). In addition, in the reproductive phase, our results show that seed production was null for all CaMV-infected accessions. This suggests that any improvement of tolerance to water deficit in the vegetative phase would have no selective advantage for the plant. By contrast, increased survival of the infected plants as found here (Fig 5), and/or increased duration of their vegetative phase, could benefit the virus through increased chance of spread in the plant population. CaMV is transmitted by insect vectors from vegetative tissues but is never disseminated via seeds [55]. It would be interesting to empirically test the evolutionary hypothesis that increased survival to abiotic stress of infected plants may be advantageous for the virus and thus would be under natural selection. This hypothesis meets the evolutionary hypothesis of virulence-transmission trade-off [56,57].

More surprisingly, we found that the survival of plants infected by CaMV was improved by water deficit. It is possible that pathways activated in response to this abiotic stress, in addition to viral infection, may allow the plant to survive longer than in the case of infection alone. Indeed, a priming effect of one stress on the other could activate similar response pathways [58], such as plant hormones [1,52,59–63], reactive oxygen species or calcium oscillation [64–67]. Water deficit may also delay, or even stop, the spread of the virus within the plant through its effects on the reduction of rates of growth and development [48].

### Ruderality may predict genotype tolerance to viral infection

As a typical ruderal species, *A*. *thaliana* is generally found in agricultural fields and other anthropogenic habitats as well as in a wide range of naturally disturbed habitats [68]. It was thus not surprising that we found a high average R-score in the Grime’s triangle CSR strategies scheme, following a previously published procedure [23]. Also, in accordance with recent findings [19,20], we found a significant variation along the R-axis across accessions. It has recently been shown that the genetic variation found in a set of 426 natural accessions of *A*. *thaliana* was associated with local climatic conditions, with evidence of adaptive selection of CSR strategies in response to climate [20]. Here, we found no significant relationships between climate at collection sites and CSR strategies. Ruderal strategies, i.e. high R scores, are typically associated with a short life-cycle, low leaf tissue density (low LDMC and/or low LMA), and presumably high metabolic rate and low tissue protection [18]. Accordingly, we found here that the ruderal character of a genotype was positively related to early bolting and flowering, and inversely to LDMC and LMA [20,27].

Our study is the first attempt to relate intraspecific variation in CSR functional strategies to virus tolerance. At the interspecific level, it has been shown that ruderal (R) species host intermediate levels of virus richness compared to competitive (C) and stress-tolerant (S) species that host a higher and a lower number of viruses, respectively [15]. The authors of the latter study suggested that this interspecific pattern could be related to the level of susceptibility to herbivores and pathogens. They also suggested that similar patterns should be observed at the intraspecific level in relation to growth strategies, as shown for fungal pathogens in radish where fast-growing populations of radish were most susceptible to *Fusarium oxysporum* [15]. On the one hand, ruderal species may be poorly tolerant to pathogens due to low levels of defensive chemicals compared to stress-tolerant (S) species [69]. On the other hand, a short life-cycle may contribute to escaping pathogen attack and may lower pathogen effects. In the case of *A*. *thaliana*, Leisner and Howell (1992) showed that early flowering accessions had a lower proportion of symptoms due to a mismatch between the kinetics of virus movement and the rate of development of the infected plant. Here, we found that the ruderal character of an accession was positively related to its capacity to tolerate virus infection, and, to a lesser extent, water deficit. Considering the adaptive value of functional strategies across the natural diversity of *A*. *thaliana* [19,20,27,43], this result affirms the importance of investigating the role of phytoviruses as selective agents in natural populations of ruderal species [36,70,71].

### Consistency of leaf trait relationships under viral infection and/or water deficit

We found similar patterns of responses to combinations of CaMV infection and watering treatment for both LDMC and LMA, two leaf traits related to leaf tissue structure and physiology. This is not surprising since these two leaf traits were highly correlated across accessions, as often reported across different scales [10,20,72]. However, it was noticed that contrasting phenotypic plasticity between these traits may translate into changes in their correlation structure under contrasting environments [72]. Here, we observed that LDMC and LMA exhibited contrasted changes in response to water deficit, with LDMC increasing more than LMA, which translated into changes in the slopes of the relationship between their responses to CaMV. High plasticity in leaf growth and development in response to water deficit is generally observed [73], and often translates into increase in leaf tissue density and leaf thickness, and therefore into increased LDMC and LMA [74,75]. Changes in leaf morphology, excluding signs of senescence, in response to viral infection have not been thoroughly described. In this study, we found that CaMV infection tended to increase both LDMC and LMA, although some accessions exhibited a different trend. These changes can impact aphid behavior and, consequently, virus transmission [76,77].

### Virus spread into the plant is affected by water deficit

It is known that the time at which viruses move out of the inoculation site into the rest of the plant varies widely depending on factors such as host and virus species, age of the host, method of inoculation and the abiotic factors of the environment [78,79]. In accordance with our previous results [13], here we showed that the lag time of symptom appearance and the rate of systemic spread of CaMV varied across accessions, and were contrastingly affected by water deficit depending on accession. As shown for other plant viruses [80], the establishment of systemic spread of the CaMV is dependent on the flow of plant metabolites via the phloem [48]. Noticeably, the pattern of visible symptoms observed in CaMV-infected *A*. *thaliana* is closely related to the pattern of viral particles spread within the plant (as detected by *in planta* hybridization of viral particles) [48]. Moreover, it has been shown that CaMV isolate Cabb B-JI, selected in our study, had similar symptom expression across the 23 *A*. *thaliana* accessions tested [81]. However, we cannot exclude differences between accessions in the lag-time between the presence of the virus in new colonization sites and the appearance of visible symptoms across the 44 accessions involved in our study. Nevertheless, by its influence on this phloem flow (i.e. carbon availability and sink organ growth influences), water deficit could influence and modify the systemic movement of viral particles [82–85].

## Conclusion

In conclusion, this work showed that intraspecific variation in the ruderal character of an *A*. *thaliana*’s genotype was positively related to its tolerance to CaMV infection, partly independently of changes due to watering conditions. This may have practical interest, as plant ecological strategies, as determined here from easily measurable traits, could be used for screening larger sets of plant genotypes and virus isolates, and for modeling purposes. In addition, contrasted plasticity between traits and/or genotype-specific susceptibility/tolerance to both virus and watering conditions may lead to deviation from this general pattern. Elucidating the evolutionary implications of increased survival to virus infection upon water deficit will require further investigation.

## Materials and methods

### Plant material and growth conditions

We selected 44 natural accessions of *A*. *thaliana* evenly located in the Iberian Peninsula and originated from a large variability of climates and altitude (S1 Table). These accessions have been sequenced by Carlos Alonso-Blanco and collaborators and belong to four distinct genetic lineages as determined by the 1001 genomes Project (http://1001genomes.org/) [86]. These accessions were grown in two experiments (experiment 1, n = 39; experiment 2, n = 20; 15 accessions in common; S1 Table) under combinations of well-watered, water deficit, mock inoculation (Mock), and CaMV-inoculation (CaMV) treatments. Experiments were conducted using the PHENOPSIS facility [47,87]. This phenotyping platform allows automated irrigation, weighing and imaging of 504 potted plants under strictly controlled environmental conditions [47]. Three to five seeds were sown at the soil surface in 225-ml pots filled with a 30:70 (v/v) mixture of clay and organic compost (substrate SP 15% KLASMANN) and placed randomly in PHENOPSIS growth chamber. Soil water content was estimated for each pot before sowing, as previously described [47]. The soil surface was moistened with deionized water, and pots were placed in the PHENOPSIS growth chamber in the dark for two days at 12°C air temperature and 70% air relative humidity. Pots were dampened with sprayed deionized water three times a day until germination. After the germination phase (*ca*. seven days), plants were cultivated under 8-h day length (experiment 1; n = 3 plants per accession and per treatment) or 12-h day length each day (experiment 2; n = 6 plants per accession and per treatment) at 200 μmol m^-2^ s^-1^ photosynthetic photon flux density, at plant height. Air temperature was set to 20°C, and air relative humidity was adjusted in order to maintain constant water vapor pressure deficit at 0.6 kPa. At the appearance of the cotyledons, one plant was kept per pot, and the temperature was set at 21/18°C day/night, while the vapor pressure deficit was set at 0.75 kPa. Each pot was weighed daily and watered with deionized water to reach the target soil relative water content. Soil relative water content was maintained at 1.4 g H_2_O g^-1^ dry soil (well-watered conditions) until application of the treatments. CaMV- (or mock) -inoculation or (see below) was performed at the emergence of the tenth rosette leaf. Water deficit was applied 1 week after inoculation the approximate timing of first symptom appearance. Irrigation of half of the CaMV- and mock-inoculated plants was stopped to reach water deficit at 0.50 H_2_O g^-1^ dry soil, reached after 7 days of water deprivation, and then maintained at this value until the end of the experiment. Under well-watered conditions, soil relative water content was maintained at 1.4 g H_2_O g^-1^ dry soil. All environmental data, including daily soil water content, air temperature, and vapor pressure deficit, are available in the PHENOPSIS database [88].

### Virus purification and plant inoculation

The CaMV, reference isolate Cabb B-JI [89]–a stylet-borne virus–was used in this study. Virus particles were purified from CaMV-infected *Brassica rapa* cv. “Just Right” (turnip) plants according to [89]. The quality and quantity of purified virus were assessed by polyacrylamide gel electrophoresis under denaturing conditions (12% SDS-PAGE) and by spectrometric measurements at 230, 260, and 280 nm (NanoDrop 2000 spectrophotometer). Virus concentration was estimated by spectrometry using the formula described by [90]. At the 10-leaf stage, *A*. *thaliana* plants were mechanically inoculated as previously described [91]. Briefly, CaMV-infected turnip extract was prepared from 1 g of infected leaf material [turnip leaves presenting systemic symptoms collected at 21 days post inoculation (dpi)] ground in 1 ml of distilled water with carborundum. Purified CaMV particles were then added to this mix at a final concentration of 0.2 mg ml^-1^ to optimize infection success. For each inoculated plant, 10 μl of the solution described above was deposited on each of three middle-rank leaves. Then, leaves were rubbed with an abrasive pestle. The control group was mock-inoculated in a similar way to mimic the wound induced by mechanical inoculation. Mock-inoculation was performed with a mix containing non-infected turnip plant extract and the buffer used for virus purification (100 mM Tris-HCl, 2.5 mM MgCl_2_, pH 7). All plants were randomly inoculated, independently of genotype and watering regime.

### Measurement of plant traits and symptom development

During the course of plant development, the following stages were scored: germination, inflorescence emergence, opening of first flower and shattering of first silique [stages 0.7, 5.01, 6.00 and 8.00 of Boyes et al. (2001), respectively].

Projected rosette area was estimated from automated daily pictures using a semi-automatic procedure developed in the image analysis environment Image J (Research Services National Institute of Mental Health, Bethesda, Maryland, USA) and downloadable from the PHENOPSIS web site.

Harvests were carried out at 30 dpi (experiment 1) or at first silique appearance (experiment 2). Each rosette (experiment 1) or one fully-expanded leaf per rosette (experiment 1 and 2) was cut, fresh mass was measured then the tissue was kept in deionized water for 24 hours at 4°C to determine the water-saturated weight (mg). A mature and non-senescent rosette leaf was marked (experiment 1) to perform all leaf measurements. After determination of the water-saturated weight, individual leaves were scanned for LA (leaf area) determination (mm^2^) using ImageJ (Research Services National Institute of Mental Health, Bethesda, Maryland, USA). Collected rosettes were oven-dried at 65°C for at least 5 days, and their dry masses determined. From these measurements, leaf dry matter content (LDMC, the ratio of dry mass to water-saturated fresh mass, mg g^–1^) and leaf mass per area (LMA, the ratio of blade dry mass to blade area, mg mm^–2^), the inverse of specific leaf area (the ratio of blade area to blade dry mass, mm^2^ mg^-1^), were calculated at the rosette (experiment 1) and leaf (experiment 1 and 2) levels as described [92].

We calculated CSR scores (i.e. percentages along the C, S and R axes) for all accessions in PHENOPSIS using the method developed by Pierce et al. (2017). The method is based on an algorithm that combines data for the three leaf traits (LA, 1/LMA and LDMC), whichwere shown to reliably position the species in the CSR scheme. CSR scores were calculated for each individual plant and per experiment with the calculation table provided in the supplementary information of [23]; the average was then calculated by genotype and experiment. CSR scores are represented in a triangle plot with the “*ggtern*” R-package [93].

Days to bolting (appearance of flower bud) and days to flowering (opening of the first flower) were calculated by reporting the bolting and flowering dates to the germination date from the second experiment data. Mature silique measurements (size of the main stem, mature siliques number) were carried out on all plants during plant harvest (experiment 2).

Relative change in values of LDMC, LMA and aboveground dry mass (ADM) was calculated for each genotype as: (mean trait value_given treatment_)–(mean trait value_mock-inoculated:WW_)*100 / (mean trait value_mock-inoculated:WW_). Tolerance was estimated for each genotype as the relative change of mean aboveground dry mass of the rosette as: (ADM_CaMV-infected:WW_−ADM_mock-inoculated:WW_)*100 / ADM_mock-inoculated:WW_) and (ADM_CaMV-infected:WD_−ADM_mock-inoculated:WW_)*100 / ADM_mock-inoculated:WW_), where WW and WD are well-watered and water deficit conditions, respectively.

Virus symptoms were monitored daily (from 9 dpi to 30 dpi) for all plants. Symptoms were scaled 0, 1 or 2 for no symptoms, presence of symptoms on a single non-inoculated leaf, or systemic symptoms, respectively. Time of symptoms appearance, rate of virus systemic spread, and maximum proportion of infected plants were then calculated from logistic regressions fitted to these observations [13]. Since it has been shown that in *A*. *thaliana* pattern of symptoms closely corresponds to the pattern of within-host spread of viral particles [48], we assumed a constant lag-time between virus colonization and appearance of visible symptoms.

Plant survival of CaMV-infected plants was estimated 30 days after germination in an additional experiment with growth conditions similar to those described in experiment 2. For each genotype, plant survival rate (%) was calculated as the ratio (number of live plants / numbers of dead plants)_._

### Data analyses

All analyses were performed in the programming environment R [94]. For each genotype, relative change was calculated as the ratio of the difference between the trait value of each replicate plant and mean trait value under control conditions (mock:well-watered) to the mean trait value under control conditions. Join hierarchical cluster analysis was performed on the relative change of aboveground dry mass in response to CaMV infection, water deficit and their combination. ANOVAs with genotype, virus infection and watering main effects and their interactive effects were performed for each trait. Tukey’s post hoc tests were performed for pairwise mean comparisons. Bootstrapped 95% confidence intervals (CI) of mean trait values were computed following the *mean_cl_boot* procedure of the Hmisc package. Nonlinear models were fitted using the *nls* function, and 95% confidence intervals for the parameters of fitted models were computed with the *confint* function of the package mass. Generalized linear models were tested using the *glm* function of the stat package. Relationships between traits were examined with Pearson’s coefficients of correlation (*r*), using the function *cor*.*test*.

The numerical data used in all figures are available here https://doi.org/10.15454/PY9KDC.

## Supporting information

S1 Fig Location and climatic conditions of the accessions collecting sites. (A) Distribution of the 44 natural accessions used in this study. Color of the points represents the altitudinal gradient. The map is shaded following the temperature gradient. (B) Mean annual precipitation and mean annual temperature for the sites where accessions were collected, in relation to major biome types of the world following Whittaker’s classification. 1–9: Tundra, Boreal forest, Temperate Grassland Desert, Woodland Shrubland, Temperate Forest, Temperate Rain Forest, Tropical Forest Savana, Tropical Rain Forest, and Desert. (C) Relationships altitude and climatic indicators at the collections sites.
(DOCX)

S2 Fig Rosette expansion rate of 39 *A*. *thaliana* accessions in control conditions at 8-h day length. Bars are mean ± 95% CI of rosette expansion rate (mm^2^ d^-1^) at 30 days post inoculation (n = 3) of plants grown in mock-inoculated:well-watered conditions.
(DOCX)

S3 Fig Least-square means for aboveground dry mass following ANOVA of 39 *A*. *thaliana* accessions grown under contrasted viral infection and watering levels. Aboveground dry mass has been log-transformed prior analysis. Bars are lsmeans ± 95% CI. for plants grown under four treatments: mock-inoculated:WW, CaMV-infected:WW, mock-inoculated:WD and CaMV-infected:WD conditions. WW: well-watered; WD: water deficit. Data are from Experiment 1.
(DOCX)

S4 Fig Relationship between relative change of aboveground dry mass (ADM) of 39 *A*. *thaliana* accessions in response to CaMV-infection and water deficit. Each point represents the mean relative change of each accession. Point colors refer to the different response groups as determined in Fig 1. Solid lines represent significant linear regressions: (A) *r* = 0.39, *P* = 0.015; (B) *r* = 0.41; *P* = 0.003. Dashed lines represent identity(1:1) line, i.e. the line of equal response. Data are from Experiment 1. WW: well-watered conditions; WD: water deficit.
(DOCX)

S5 Fig Relationship between R-score and relative change of aboveground dry mass (%) in response to CaMV-infection and water deficit for five response groups. Each point represents a response group as determined in Fig 1. Plants were CaMV-infected or mock-inoculated under well-watered (WW) and water deficit (WD) conditions Colors are similar to response groups I-IV in Fig 1. Lines represent significant linear regressions.
(DOCX)

S6 Fig Effects of CaMV infection and watering on leaf mass per area (LMA) of 39 *A*. *thaliana* accessions. (A) Bars are means ± 95% CI of leaf mass per area (LMA; mg mm^-2^) at 30 dpi (n = 3) of plants grown in mock-inoculated:WW (white bars), CaMV-infected:WW (dark grey bars), mock-inoculated:WD (light grey bars) and CaMV-infected:WD (black bars) conditions. (B) Relative change (± se) of LMA (%) in mock-inoculated:WW (white circle), CaMV-infected:WW (dark grey triangle), mock-inoculated:WD (light grey diamond) and CaMV-infected:WD (black square) conditions. Data are from Experiment 1.
(DOCX)

S7 Fig Effects of CaMV infection and watering on leaf dry matter content (LDMC) of 39 *A*. *thaliana* accessions. (A) Bars are means ± 95% CI of leaf dry matter content (LDMC; mg g^-1^) at 30 dpi (n = 3) of plants grown in mock-inoculated:WW (white bars), CaMV-infected:WW (dark grey bars), mock-inoculated:WD (light grey bars) and CaMV-infected:WD (black bars) conditions. (B) Relative change (± se) of LDMC (%) in mock-inoculated:WW (white circle), CaMV-infected:WW (dark grey triangle), mock-inoculated:WD (light grey diamond) and CaMV-infected:WD (black square) conditions. Data are from Experiment 1.
(DOCX)

S8 Fig Comparison of the relationships between LMA and LDMC and inherent growth rates at 8-h day length and 12-h day length. (A) Relationship between leaf mass per area (LMA; mg mm^-2^) and leaf dry matter content (LDMC; mg g^-1^) in experiment 1. (B) Relationship between LMA and LDMC in experiment 2. Each point represents the mean relative change of each genotype. Lines represent significant linear regressions (*P* < 0.001). Mock-inoculated:WW (white circle), CaMV-infected:WW (dark grey triangle), mock-inoculated:WD (light grey diamond) and CaMV-infected:WD (black square). Data are from Experiment 1 and 2 (n = 39, 8-h day length and n = 20, 12-h day length, respectively). (C) Relationship between expansion rate (mm^2^ d^-1^) at 8-h and 12-h day length on 15 *A*. *thaliana* accessions (*r* = 0.71, P = 0.003).
(DOCX)

S9 Fig Relationship between vegetative growth and leaf morphological traits of 39 *A*. *thaliana* accessions. Each point represents an accession under well-watered (WW; dark blue circle) or water deficit (WD; light blue triangle) conditions. (A) Relationship between relative change of aboveground dry mass production (ADM; %) in CaMV-infected plants and leaf dry matter content (LDMC; mg g^-1^; Pearson’s *r* = –0.43, *P* < 0.007 for WW and *r* = –0.20, *P* = 0.23 for WD). (B) Relationship between relative change of ADM (%) in CaMV infected plants under WW and under WD and leaf mass per area (LMA; mg mm^-2^; *r* = –0.52, *P* < 0.001 for WW and *r* = –0.35, *P* = 0.031 for WD). Lines represent significant linear regressions at *P* < 0.05. Data are from Experiment 1.
(DOCX)

S10 Fig Effects of CaMV infection and watering treatment on phenological traits of 20 *A*. *thaliana* accessions. (A) Days to bolting. (B) Days to flowering. Bars and error bars are means ± bootstrapped 95% confidence intervals of plants grown under mock-inoculated:WW (white bars), mock-inoculated:WD (dark grey bars), CaMV-infected:WW (light grey bars) and CaMV-infected:WD (black bars) conditions. WW: well-watered conditions; WD: water deficit Accessions are ordered according to their final projected area of the rosette in condition of viral infection. Data are from Experiment 2.
(DOCX)

S11 Fig Relationships between leaf morphological traits, ruderality and phenology of 20 accessions of *A*. *thaliana*. Relationships of days to bolting vs. (A) leaf dry matter content (LDMC; mg g-1), (B) leaf mass per area (LMA; mg mm-2), and (C) ruderal score (R; %), and days to flowering vs. (D) LDMC, (E) LMA, and (F) R. Each point represents an accession grown under the control condition (well-watered x mock-inoculation). Lines are significant linear regressions at *P* < 0.05. Data are from Experiment 2.
(DOCX)

S12 Fig Dynamics of viral symptoms in 20 accessions of *A*. *thaliana* infected with CaMV under well-watered and water deficit conditions. (A) Lag time of symptoms appearance. (B) Rate of symptoms appearance. Bars and error bars are means ± 95% confidence intervals extracted from sigmoidal curve fitting of symptom dynamics under well-watered (grey bars) and water deficit (black bars) conditions. Marks above the bars indicate 5%-level significant decrease (–) or increase (+) in response to the water deficit. Accessions are ordered according to increasing final projected area of the rosette. Data are from Experiment 2.
(DOCX)

S1 Table Geographic origin and location of Iberian *A*. *thaliana* accessions used in this study. (DOCX)

S2 Table ANOVA for aboveground dry mass (log_10_), leaf dry matter content (LDMC; log_10_) and leaf mass per area (LMA; log_10_) for 39 *A*. *thaliana*’s accessions grown under well-watered and water deficit conditions and mock- or CaMV-inoculated. (DOCX)

S3 Table ANCOVA of the relationships between LMA and LDMC at 8-h or 12-h day length. (DOCX)
