# Supplementary material for: Natural variation of Arabidopsis thaliana responses to Cauliflower mosaic virus infection upon water deficit
Source: PLoS Pathog. 2020 May 15;16(5):e1008557. doi: 10.1371/journal.ppat.1008557 (PMC7255604; doi:10.1371/journal.ppat.1008557)
Supplement: S8 Fig — (A) Relationship between leaf mass per area (LMA; mg mm-2) and leaf dry matter content (LDMC; mg g-1) in experiment 1. (B) Relationship between LMA and LDMC in experiment 2. Each point represents the mean relative change of each genotype. Lines represent significant linear regressions (P < 0.001). Mock-inoculated:WW (white circle), CaMV-infected:WW (dark grey triangle), mock-inoculated:WD (light grey diamond) and CaMV-infected:WD (black square). Data are from Experiment 1 and 2 (n = 39, 8-h day length and n = 20, 12-h day length, respectively). (C) Relationship between expansion rate (mm2 d-1) at 8-h and 12-h day length on 15 A. thaliana accessions (r = 0.71, P = 0.003). (DOCX) [file ppat.1008557.s008.docx]

**S8 Fig.**
