# Supplementary material for: Natural variation of Arabidopsis thaliana responses to Cauliflower mosaic virus infection upon water deficit
Source: PLoS Pathog. 2020 May 15;16(5):e1008557. doi: 10.1371/journal.ppat.1008557 (PMC7255604; doi:10.1371/journal.ppat.1008557)
Supplement: S9 Fig — Each point represents an accession under well-watered (WW; dark blue circle) or water deficit (WD; light blue triangle) conditions. (A) Relationship between relative change of aboveground dry mass production (ADM; %) in CaMV-infected plants and leaf dry matter content (LDMC; mg g-1; Pearson’s r = –0.43, P < 0.007 for WW and r = –0.20, P = 0.23 for WD). (B) Relationship between relative change of ADM (%) in CaMV infected plants under WW and under WD and leaf mass per area (LMA; mg mm-2; r = –0.52, P < 0.001 for WW and r = –0.35, P = 0.031 for WD). Lines represent significant linear regressions at P < 0.05. Data are from Experiment 1. (DOCX) [file ppat.1008557.s009.docx]

**S9 Fig.**
