# Supplementary material for: Natural variation of Arabidopsis thaliana responses to Cauliflower mosaic virus infection upon water deficit
Source: PLoS Pathog. 2020 May 15;16(5):e1008557. doi: 10.1371/journal.ppat.1008557 (PMC7255604; doi:10.1371/journal.ppat.1008557)
Supplement: S10 Fig — (A) Days to bolting. (B) Days to flowering. Bars and error bars are means ± bootstrapped 95% confidence intervals of plants grown under mock-inoculated:WW (white bars), mock-inoculated:WD (dark grey bars), CaMV-infected:WW (light grey bars) and CaMV-infected:WD (black bars) conditions. WW: well-watered conditions; WD: water deficit Accessions are ordered according to their final projected area of the rosette in condition of viral infection. Data are from Experiment 2. (DOCX) [file ppat.1008557.s010.docx]

**S10 Fig.**
