# Supplementary material for: Natural variation of Arabidopsis thaliana responses to Cauliflower mosaic virus infection upon water deficit
Source: PLoS Pathog. 2020 May 15;16(5):e1008557. doi: 10.1371/journal.ppat.1008557 (PMC7255604; doi:10.1371/journal.ppat.1008557)
Supplement: S11 Fig — Relationships of days to bolting vs. (A) leaf dry matter content (LDMC; mg g-1), (B) leaf mass per area (LMA; mg mm-2), and (C) ruderal score (R; %), and days to flowering vs. (D) LDMC, (E) LMA, and (F) R. Each point represents an accession grown under the control condition (well-watered x mock-inoculation). Lines are significant linear regressions at P < 0.05. Data are from Experiment 2. (DOCX) [file ppat.1008557.s011.docx]

**S11 Fig**
