# Supplementary material for: Natural variation of Arabidopsis thaliana responses to Cauliflower mosaic virus infection upon water deficit
Source: PLoS Pathog. 2020 May 15;16(5):e1008557. doi: 10.1371/journal.ppat.1008557 (PMC7255604; doi:10.1371/journal.ppat.1008557)
Supplement: S12 Fig — (A) Lag time of symptoms appearance. (B) Rate of symptoms appearance. Bars and error bars are means ± 95% confidence intervals extracted from sigmoidal curve fitting of symptom dynamics under well-watered (grey bars) and water deficit (black bars) conditions. Marks above the bars indicate 5%-level significant decrease (–) or increase (+) in response to the water deficit. Accessions are ordered according to increasing final projected area of the rosette. Data are from Experiment 2. (DOCX) [file ppat.1008557.s012.docx]

**S12 Fig.**
