# Supplementary material for: Natural variation of Arabidopsis thaliana responses to Cauliflower mosaic virus infection upon water deficit
Source: PLoS Pathog. 2020 May 15;16(5):e1008557. doi: 10.1371/journal.ppat.1008557 (PMC7255604; doi:10.1371/journal.ppat.1008557)
Supplement: S1 Table — (DOCX) [file ppat.1008557.s013.docx]

**S1 Table. Geographic origin and location of Iberian *A. thaliana* accessions used in this study.**

| id 1001 genome | Accession name | Country | Latitude | Longitude | Genetic group | Experience |
| --- | --- | --- | --- | --- | --- | --- |
| 9515 | Ala-0 | ESP | 39,72 | -6,89 | Spain | 1 & 2 |
| 9519 | Ang-0 | ESP | 41,94 | 2,64 | Spain | 1 |
| 9820 | Are-0 | ESP | 41 | -4,71 | Spain | 1 |
| 9822 | Aul-0 | ESP | 40,52 | -4,02 | Spain | 1 |
| 9522 | Bea-0 | ESP | 36,52 | -5,27 | Spain | 2 |
| 8264 | Bla-1 | ESP | 41,6833 | 2,8 | Spain | 1 & 2 |
| 9825 | Boa-0 | ESP | 40,4 | -3,88 | Spain | 1 |
| 9827 | Bos-0 | ESP | 42,78 | 0,69 | Western Europe | 1 & 2 |
| 7063 | Can-0 | ESP | 29,2144 | -13,4811 | Relict | 2 |
| 9943 | Cdm-0 | ESP | 39,73 | -5,74 | Spain | 1 & 2 |
| 9834 | Cho-0 | ESP | 40,51 | -3,9 | Spain | 1 |
| 7081 | Co | POR | 40,2077 | -8,42639 | Spain | 1 |
| 9507 | Coa-0 | POR | 38,45 | -7,5 | Spain | 2 |
| 6909 | Col-0 | USA | 38,3 | -92,3 | Germany | 1 & 2 |
| 9838 | Cot-0 | ESP | 41,83 | -5,38 | Admixed | 1 |
| 9841 | Ees-0 | ESP | 40,59 | -4,15 | Spain | 1 |
| 9844 | Esn-2 | ESP | 42,27 | 0,19 | Spain | 1 |
| 9941 | Fei-0 | POR | 40,92 | -8,54 | Western Europe | 2 |
| 9847 | Fel-2 | ESP | 43,31 | -5,7 | Western Europe | 1 & 2 |
| 9544 | Gua-1 | ESP | 39,4 | -5,33 | Spain | 1 & 2 |
| 9852 | Ini-0 | ESP | 40,46 | -3,75 | Spain | 1 |
| 9853 | Lac-0 | ESP | 43,33 | -5,91 | Western Europe | 1 |
| 9855 | Lam-0 | ESP | 40,57 | -3,89 | Spain | 1 & 2 |
| 9856 | Lch-0 | ESP | 40,51 | -4 | Spain | 1 |
| 9864 | Mat-0 | ESP | 41,76 | 2,69 | Spain | 1 |
| 9868 | Moe-0 | ESP | 41,78 | 2,37 | Spain | 1 & 2 |
| 9873 | Ndc-0 | ESP | 37,94 | -5,45 | Spain | 1 |
| 9565 | Orb-10 | ESP | 42,97 | -1,23 | Admixed | 1 & 2 |
| 9875 | Ovi-1 | ESP | 43,38 | -5,87 | Western Europe | 1 & 2 |
| 9879 | Per-0 | ESP | 37,6 | -1,12 | Relict | 1 & 2 |
| 9883 | Piq-0 | ESP | 42,1 | -2,56 | Spain | 1 & 2 |
| 9888 | Pva-1 | ESP | 40,93 | -3,31 | Spain | 1 |
| 9573 | Rds-0 | ESP | 41,86 | 2,99 | Spain | 1 |
| 9510 | Rei-0 | POR | 38,75 | -7,59 | Spain | 1 |
| 9578 | Sac-0 | ESP | 42,13 | -6,7 | Western Europe | 1 |
| 6961 | Se-0 | ESP | 38,3333 | -3,53333 | Spain | 1 |
| 7328 | Sf-2 | ESP | 41,7833 | 3,03333 | Spain | 1 & 2 |
| 9895 | Sfb-6 | ESP | 41,78 | 2,57 | Spain | 1 |
| 9899 | Tau-0 | ESP | 42,54 | 0,84 | Spain | 1 |
| 9588 | Tol-7 | ESP | 42,11 | 0,6 | Spain | 1 |
| 9591 | Vad-0 | ESP | 42,86 | -3,59 | Admixed | 2 |
| 9512 | Vid-1 | POR | 38,22 | -7,84 | Spain | 1 |
| 9597 | Vig-1 | ESP | 42,31 | -2,53 | Spain | 1 & 2 |
| 9599 | Vin-0 | ESP | 42,8 | -5,77 | Western Europe | 1 |
