# Supplementary material for: Natural variation of Arabidopsis thaliana responses to Cauliflower mosaic virus infection upon water deficit
Source: PLoS Pathog. 2020 May 15;16(5):e1008557. doi: 10.1371/journal.ppat.1008557 (PMC7255604; doi:10.1371/journal.ppat.1008557)
Supplement: S2 Table — (DOCX) [file ppat.1008557.s014.docx]

**S2 Table. ANOVA for aboveground dry mass (log_10_), leaf dry matter content (LDMC; log_10_) and leaf mass per area (LMA; log_10_) for 39 *A. thaliana*’s accessions grown under well-watered and water deficit conditions and mock- or CaMV-inoculated.**

| **Trait** | **Effect** | **df** | **F** | **P-value** |
| --- | --- | --- | --- | --- |
| Aboveground dry mass | Accession | 38 | 7.08 | < 0.001 |
|  | Inoculation | 1 | 159.62 | < 0.001 |
|  | Watering | 1 | 381.44 | < 0.001 |
|  | Acc*Inoculation | 38 | 1.54 | 0.02 |
|  | Acc*Watering | 38 | 1.00 | 0.47 |
|  | Inoculation*Watering | 1 | 0.84 | 0.36 |
|  | Acc*Inoculation*Watering | 38 | 0.94 | 0.58 |
|  |  |  |  |  |
| LDMC | Accession | 38 | 3.68 | < 0.001 |
|  | Inoculation | 1 | 33.35 | < 0.001 |
|  | Watering | 1 | 10.74 | < 0.001 |
|  | Acc*Inoculation | 38 | 1.32 | 0.01 |
|  | Acc*Watering | 38 | 0.69 | 0.03 |
|  | Inoculation*Watering | 1 | 0.02 | 0.93 |
|  | Acc*Inoculation*Watering | 38 | 1.07 | 0.06 |
|  |  |  |  |  |
| LMA | Accession | 38 | 3.68 | < 0.001 |
|  | Inoculation | 1 | 33.35 | < 0.001 |
|  | Watering | 1 | 10.74 | 0.001 |
|  | Acc*Inoculation | 38 | 1.32 | 0.11 |
|  | Acc*Watering | 38 | 0.69 | 0.91 |
|  | Inoculation*Watering | 1 | 0.02 | 0.01 |
|  | Acc*Inoculation*Watering | 38 | 1.07 | 0.37 |
