# Supplementary material for: Natural variation of Arabidopsis thaliana responses to Cauliflower mosaic virus infection upon water deficit
Source: PLoS Pathog. 2020 May 15;16(5):e1008557. doi: 10.1371/journal.ppat.1008557 (PMC7255604; doi:10.1371/journal.ppat.1008557)
Supplement: S3 Table — (DOCX) [file ppat.1008557.s015.docx]

**S3 Table. ANCOVA of the relationships between LMA and LDMC at 8-h or 12-h day length.**

|  | **LMA at-8-h photoperiod** | | | **LMA at 12-h photoperiod** | | |
| --- | --- | --- | --- | --- | --- | --- |
| *Predictors* | *Estimates* | *p* | *Estimates* | | *p* |  |
| Intercept | 142.65 | **<0.001** | 62.67 | | **<0.001** |  |
| LDMC | -1.05 | **<0.001** | -0.18 | | **<0.001** |  |
| Inoculation (I) | -17.61 | 0.092 | -6.90 | | 0.114 |  |
| Watering (W) | -19.62 | 0.091 | -5.55 | | 0.262 |  |
| LDMC:I | 0.26 | 0.067 | 0.03 | | 0.159 |  |
| LDMC:W | 0.31 | 0.043 | 0.03 | | 0.221 |  |
| I:W | 0.54 | 0.971 | -1.25 | | 0.866 |  |
| LDMC:I:W | -0.05 | 0.793 | 0.01 | | 0.817 |  |
| Observations | 156 |  | 79 | |  |  |
| R^2^ | **0.705** | **<0.001** | **0.805** | | **<0.001** |  |
|  |  |  |  | |  |  |
